# Editorial: Improving the quality of life of autistic people and their caregivers from diverse backgrounds: methods and approaches

**DOI:** 10.3389/fpsyg.2023.1242236

**Published:** 2023-11-13

**Authors:** Harish Katti, Georgitta Valiyamattam, Jessica Taubert, Aparna Nadig

**Affiliations:** ^1^National Institute of Mental Health, Bethesda, MD, United States; ^2^The Department of Applied Psychology, Gitam University, Visakhapatnam, India; ^3^The School of Psychology, The University of Queensland, St. Lucia, QLD, Australia; ^4^School of Communication Sciences and Disorders, McGill University, Montreal, QC, Canada

**Keywords:** autism, caregiving, quality of life, diversity and inclusion, culture

Across cultures, caregivers and the immediate family are often the primary sources of referral for developmental atypicalities such as the symptoms of autism spectrum conditions. This becomes particularly relevant in low- and middle-income economies where the onus of early identification relies heavily on caregiver awareness (Elsabbagh et al., 2012). In the DSM-V definition, individuals with autism spectrum disorder require additional support, Level 1 support, Level 2 substantial support, or Level 3 very substantial support in each of the core symptom areas of Social Communication and Restricted and Interests & Repetitive Behaviors (American Psychiatric Association, 2013) Consequently, the extent of caregiving or support required is modulated by the severity of autism symptoms, as well as the symptoms of co-occurring conditions (Salomone et al., 2018). The areas of caregiving can include, but are not limited to, facilitating daily living and self-care activities, while also supporting communication and other cognitive and socioemotional tasks. Ongoing, intensive care throughout the lifespan is required for a significant proportion of autistic individuals. Due to the pervasiveness of the care-giving role, families with an autistic individual are often referred to as families living with autism (Sivberg, 2002; Neely-Barnes et al., 2011). While the role played by the caregivers is crucial, and can have positive outcomes for the caregivers, it is necessary to acknowledge that it is associated with several challenges. A substantial amount of literature reflects the physical, financial and emotional toll that the diagnosis of autism spectrum conditions brings to the caregivers resulting in less leisure, chronic stress, and increased conflict with an overall decrease in quality of life (QoL) (e.g., Lavelle et al., 2014; Hartley et al., 2017; Pisula and Porêbowicz-Dörsmann, 2017; Al Khateeb et al., 2019; Rogge and Janssen, 2019; Walton, 2019). In fact, research findings suggest that levels of parenting stress associated with autism spectrum conditions is higher compared to other developmental disabilities (Hayes and Watson, 2013; Cohrs and Leslie, 2017).

Within the context of caregiving in autism, two aspects deserve a special mention; Firstly, while it is accepted that cultural differences exist in how developmental disorders are conceptualized and managed, there is a dearth of studies examining parenting roles and concerns across cultures. A majority of studies in this domain focus on the western context and on the white-euro-American population, which limits understanding of caregiving experiences in non-western or multicultural contexts (Daley, 2004). Secondly, even with the references to *families* living with autism, the majority of research on caregiving has focused specifically on mothers (Gavidia-Payne and Stoneman, 2004; Bailey and Powell, 2005). Such a trend not only leads to an obscuring of the experiences of other family members, particularly other parents, but also indirectly strengthens the narrative of mothers being the primary caregivers, thus also adding to the expectations required from the maternal role.

The five papers included in this special issue directly and indirectly allude to these themes. The first paper focuses on the role played by parents or family members or friends, who are often non-specialists, in the early identification and management of autism. In one of the first studies of its kind in a Latin American setting, Buffle et al., explore how the early diagnostic markers of autism are perceived and explained by the general adult population in Ecuador. Findings suggest that several of the core autism symptoms, with the exception of language impairment, are not considered to be a concern or related to a developmental disorder by the majority of parents, with explanations for the behavior often emphasizing the child's personality. Attitudes toward certain core symptoms in the Ecuadorian context (an upper-middle resource setting) also did not completely align with those from other low and middle resource settings, where the same symptoms (for e.g., avoiding eye-contact) were perceived as concerning. This indicates that the impact of cultural and socio-demographic factors in defining acceptable/non-acceptable behaviors may be more unique and less generalizable than previously imagined. The study underlines the need for continued education informed by research, to aid early identification, help seeking and a change in attitude toward autistic individuals.

In the second paper, Xu et al., investigate caregiver contributions to language acquisition in Mandarin-exposed typically developing children and language-matched autistic children. Building on recent research findings of positive contributions of caregiver speech in the acquisition of the English language, the study examines whether a similar relationship exists in the case of word order acquisition in Mandarin. While the abilities of the children (in terms of diagnostic group membership) are seen to modulate some aspects of word order acquisition, for instance stereotyped speech in the case of multiverb utterances, other aspects were found to be preserved irrespective of the diagnosis, including similar rates of input from the caregivers. These findings attest to the role played by caregivers in general language development particularly the length and complexity of the sentences used (and not the finer aspects of specific word order acquisition). These detailed findings can aid potential longitudinal examinations of caregiver contributions to language development in autism spectrum conditions.

The three other studies that form a part of this special issue contextualize the inter-related aspects of the quality of life of caregivers of children on the autism spectrum, when taking culture into consideration. The first study addresses cultural differences in caregiver quality of life, an area worthy of examination, as mentioned earlier. Eapen et al. interview caregivers and parents from seven countries to evaluate the QoL of parents of persons with autism spectrum conditions, as well as the impact of socio-cultural contexts in the respective country. The overall assessment of this study is that despite a consistent understanding of the nature of autism and its effects on the lives of affected individuals, the impact on parental wellbeing and QoL is variable and significantly influenced by cultural context. The study also highlights the complexities of designing public policy and health care infrastructure to support persons with autism spectrum conditions and their caregivers. This can be seen in the diverse range of scores achieved by different countries and the fact that no country achieved all goals related to support for persons with autism spectrum conditions and their caregivers. This work could be a valuable resource for reframing effective health care policies.

Social support is widely believed to enhance overall wellbeing and QoL in caregivers of individuals with disabilities. However, several additional factors may determine what kind of social support is most effective- for e.g., perceived vs. received social support or informal vs. formal vs. reciprocal social support (Hogan et al., 2002). Against this backdrop, Bi et al. examine the structure of the social support network, the levels of perceived social support and its indirect effects on subjective wellbeing in mothers of autistic children within the Chinese cultural context. Both the size of the social support network and the degree of intimacy received were seen to influence the perceived effectiveness of different social support domains. When support came from more formal sources (such as teachers or other parents) the frequency of contact was vital, whereas in the case of support being provided by family or close friends, the size of the network was vital. These insights become valuable in terms of providing effective social support, both in terms of how it is perceived and in terms of the actual needs being met.

The complexities that emerge in caregiving for adolescents and adults with autism spectrum conditions have rarely been a subject of research focus. This is especially true in the case of designing interventions for building self-efficacy in caregivers, as to aid in a sense of resilience and empowerment that becomes essential in long-term caregiving (Marsack-Topolewski et al., 2021). In the last paper in this Research Topic, Leung et al. try to fill this gap by examining the efficacy of the Core Autism Parenting Skills (CAPS) in parents of autistic adults. This culturally-sensitive, group intervention program, based in Hong Kong, seeks to improve competencies in four critical parental roles; specifically, to observe, to reinforce, to empathize and to accompany. Results indicate enhanced levels of parental self-efficacy in addition to a greater appreciation by caregivers of themselves and their needs. Considering the often-complicated equation of permitting autonomy vs. intervening, when parenting young adults, the CAPS underscores the importance of observing before deciding to intervene. This work underscores the advantages associated with the development of a culturally adapted intervention program targeting parental competence, and one that is specifically catered to the needs of an underserved population i.e., parents of autistic adults.

The articles in this Research Topic, individually and collectively seek to enable a better understanding of caregiver roles and experiences, within understudied cultural contexts. On a positive note, four of these studies with a focus on caregiver opinions and inputs, quality of life and intervention efficacy, include both maternal and paternal experiences. These studies, while forming the groundwork for further research in their respective domains (both with respect to topic and geographically), also point to the need for more research on caregiver experiences that account for cultural variables and tailored interventions that draw on cultural awareness. We therefore hope that this Research Topic at a broader level serves as a means of highlighting the need for multicultural autism research.

## Author contributions

HK, GV, AN, and JT reviewed the papers included in the Research Topic, prepared, and reviewed the editorial manuscript. All authors agreed on the final version of the editorial.
